# Biannual mass azithromycin distributions and malaria parasitemia in pre-school children in Niger: A cluster-randomized, placebo-controlled trial

**DOI:** 10.1371/journal.pmed.1002835

**Published:** 2019-06-25

**Authors:** Ahmed M. Arzika, Ramatou Maliki, Nameywa Boubacar, Salissou Kane, Sun Y. Cotter, Elodie Lebas, Catherine Cook, Robin L. Bailey, Sheila K. West, Philip J. Rosenthal, Travis C. Porco, Thomas M. Lietman, Jeremy D. Keenan, for the MORDOR Study Group

**Affiliations:** 1 The Carter Center, Niamey, Niger; 2 Francis I Proctor Foundation, University of California, San Francisco, San Francisco, California, United States of America; 3 London School of Hygiene and Tropical Medicine, London, United Kingdom; 4 Dana Center for Preventive Ophthalmology, Johns Hopkins University, Baltimore, Maryland, United States of America; 5 Department of Medicine, University of California, San Francisco, San Francisco, California, United States of America; 6 Department of Ophthalmology, University of California, San Francisco, San Francisco, California, United States of America; 7 Department of Epidemiology & Biostatistics, University of California, San Francisco, San Francisco, California, United States of America; 8 Institute for Global Health Sciences, University of California, San Francisco, San Francisco, California, United States of America; Mahidol-Oxford Tropical Medicine Research Unit, THAILAND

## Abstract

**Background:**

Mass azithromycin distributions have been shown to reduce mortality in preschool children, although the factors mediating this mortality reduction are not clear. This study was performed to determine whether mass distribution of azithromycin, which has modest antimalarial activity, reduces the community burden of malaria.

**Methods and findings:**

In a cluster-randomized trial conducted from 23 November 2014 until 31 July 2017, 30 rural communities in Niger were randomized to 2 years of biannual mass distributions of either azithromycin (20 mg/kg oral suspension) or placebo to children aged 1 to 59 months. Participants, field staff, and investigators were masked to treatment allocation. The primary malaria outcome was the community prevalence of parasitemia on thick blood smear, assessed in a random sample of children from each community at study visits 12 and 24 months after randomization. Analyses were performed in an intention-to-treat fashion. At the baseline visit, a total of 1,695 children were enumerated in the 15 azithromycin communities, and 3,029 children were enumerated in the 15 placebo communities. No communities were lost to follow-up. The mean prevalence of malaria parasitemia at baseline was 8.9% (95% CI 5.1%–15.7%; 52 of 552 children across all communities) in the azithromycin-treated group and 6.7% (95% CI 4.0%–12.6%; 36 of 542 children across all communities) in the placebo-treated group. In the prespecified primary analysis, parasitemia was lower in the azithromycin-treated group at month 12 (mean prevalence 8.8%, 95% CI 5.1%–14.3%; 51 of 551 children across all communities) and month 24 (mean 3.5%, 95% CI 1.9%–5.5%; 21 of 567 children across all communities) than it was in the placebo-treated group at month 12 (mean 15.3%, 95% CI 10.8%–20.6%; 81 of 548 children across all communities) and month 24 (mean 4.8%, 95% CI 3.3%–6.4%; 28 of 592 children across all communities) (*P* = 0.02). Communities treated with azithromycin had approximately half the odds of parasitemia compared to those treated with placebo (odds ratio [OR] 0.54, 95% CI 0.30 to 0.97). Parasite density was lower in the azithromycin group than the placebo group at 12 and 24 months (square root–transformed outcome; density estimates were 7,540 parasites/μl lower [95% CI −350 to −12,550 parasites/μl; *P* = 0.02] at a mean parasite density of 17,000, as was observed in the placebo arm). No significant difference in hemoglobin was observed between the 2 treatment groups at 12 and 24 months (mean 0.34 g/dL higher in the azithromycin arm, 95% CI −0.06 to 0.75 g/dL; *P* = 0.10). No serious adverse events were reported in either group, and among children aged 1 to 5 months, the most commonly reported nonserious adverse events (i.e., diarrhea, vomiting, and rash) were less common in the azithromycin-treated communities. Limitations of the trial include the timing of the treatments and monitoring visits, both of which took place before the peak malaria season, as well as the uncertain generalizability to areas with different malaria transmission dynamics.

**Conclusions:**

Mass azithromycin distributions were associated with a reduced prevalence of malaria parasitemia in this trial, suggesting one possible mechanism for the mortality benefit observed with this intervention.

**Trial registration:**

The trial was registered on ClinicalTrials.gov (NCT02048007).

## Abstract 2

Jeremy Keenan and colleagues study the implications of mass azithromycin treatment for malaria outcomes.

## Author summary

**Why was this study done?:**

- The Macrolides Oraux pour Réduire les Décés avec un Oeil sur la Resistance (MORDOR) trial found that distributing the antibiotic azithromycin to all preschool children in communities in Niger was effective for preventing childhood mortality.
- It is unclear how azithromycin might prevent mortality, though this antibiotic has moderate activity against malaria parasites.

**What did the researchers do and find?:**

- Thirty communities from the same study area as MORDOR were randomized to receive the same interventions: either mass distribution of azithromycin or mass distribution of placebo to all children ages 1 to 59 months every 6 months over a 2-year period.
- A blood smear test for malaria was performed on a random sample of 40 children per community before treatment and at 12 and 24 months after the initial distribution.
- Communities randomized to the antibiotic had fewer malaria parasites at months 12 and 24 than those randomized to placebo.

**What do these findings mean?:**

- Mass azithromycin distributions may reduce the burden of malaria in Niger, which may in turn contribute to the mortality benefit of this intervention.
- Future studies will be important to study how frequently to give treatment, the effectiveness of azithromycin during various seasons of the year, and the generalizability of this finding to other settings in sub-Saharan Africa.

## Introduction

Mass azithromycin distributions have lowered childhood mortality in Africa, although the mechanism explaining this effect is unknown. Macrolides Oraux pour Réduire les Décés avec un Oeil sur la Resistance (MORDOR) was a cluster-randomized placebo-controlled trial conducted in Malawi, Niger, and Tanzania that found a 14% reduction in childhood mortality in communities randomized to biannual mass azithromycin distributions targeted to 1- to 59-month-old children. The protective effect of azithromycin distributions was especially high in Niger, where malaria accounts for a large proportion of childhood deaths [1]. Azithromycin has activity against the malarial apicoplast and has demonstrated modest antimalarial activity in vitro and in numerous field studies [2–13]. Thus, the mortality benefit observed with mass azithromycin distributions may be due, in part, to decreased malaria.

MORDOR was designed as a large simple trial the outcome of which was childhood mortality as assessed on biannual census. Detailed health assessments were not performed in MORDOR in order to minimize co-interventions that could have biased the result of the trial. Instead, additional communities drawn from the MORDOR study area were enrolled into a parallel trial with the same interventions that also included annual monitoring visits to investigate possible mechanisms by which azithromycin impacted mortality. The present report details the results of blood smears processed for malaria in the parallel trial from Niger. Cluster randomization was employed to account for both the direct effects of the antibiotic as well as any indirect spillover effects of widespread community antibiotic use [14]. We hypothesized that mass azithromycin distributions would reduce the community prevalence of malaria parasitemia relative to placebo.

## Methods

### Trial design

**Fig 1:**
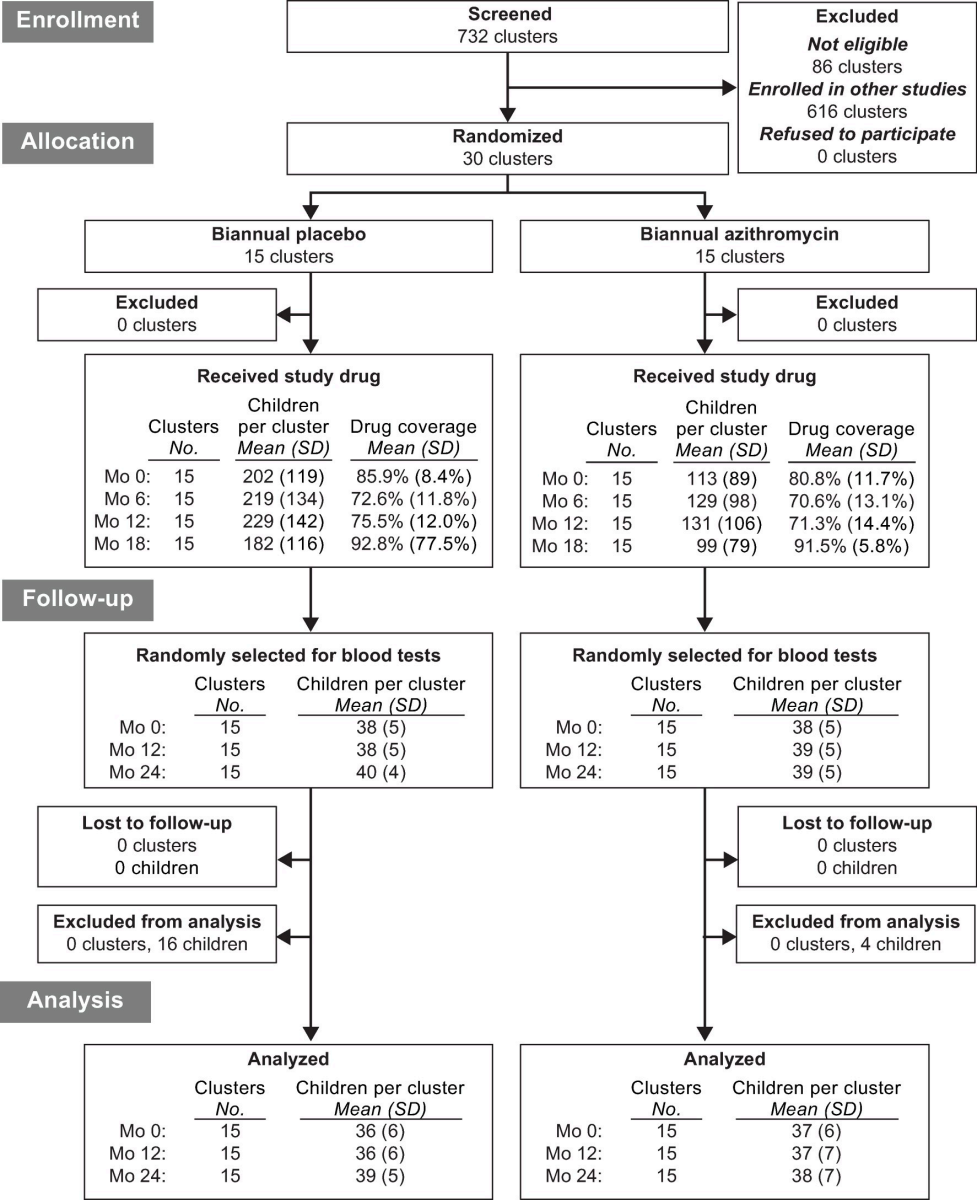
Trial flow. Communities were selected from the same pool of communities as the parent MORDOR trial; communities were excluded before randomization if not in the desired population range (i.e., 200–2,000 people) or if not required to reach the targeted sample size. A census was conducted approximately every 6 months. A random sample of children was selected at months 0, 12, and 24 for monitoring. Children excluded from analysis had thick smears that were missing or unreadable. MORDOR, Macrolides Oraux pour Réduire les Décés avec un Oeil sur la Resistance.

**Fig 2:**
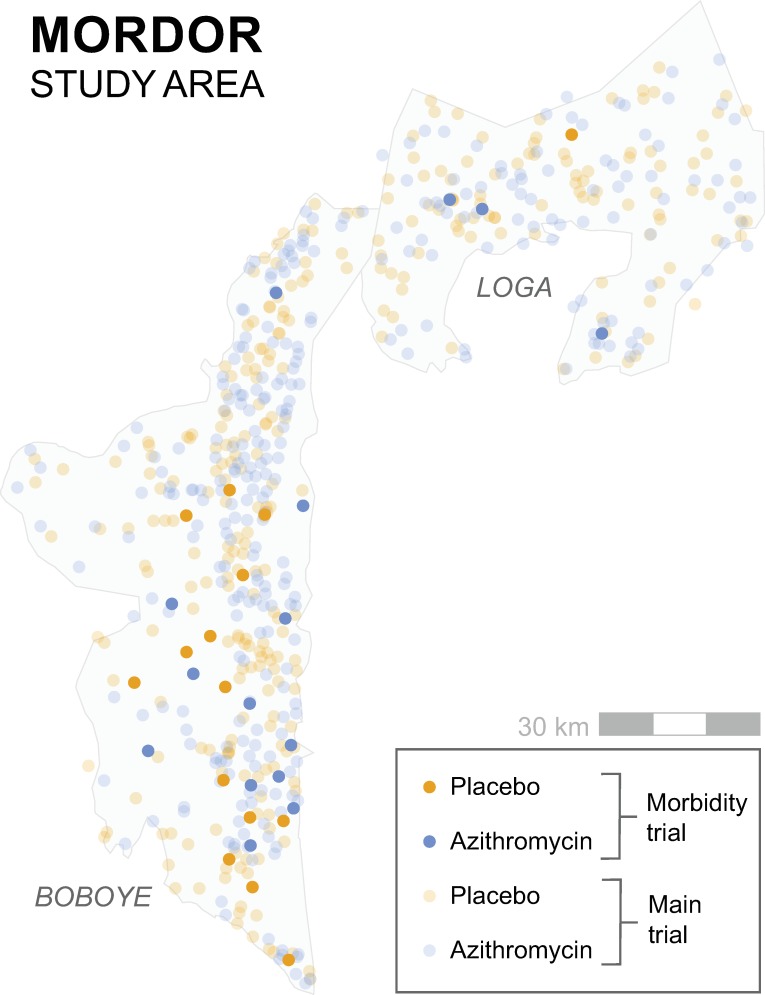
MORDOR study area. MORDOR was conducted in Boboye and Loga departments, Dosso region, Niger. Each point represents a community, with azithromycin-treated communities in blue and placebo-treated communities in orange. The 30 communities enrolled in the present study (darker markers) were randomly selected from the same pool of communities as the main MORDOR trial (lighter markers). Base maps of the Loga and Boboye departments were taken from the Humanitarian Data Exchange (https://data.humdata.org). MORDOR, Macrolides Oraux pour Réduire les Décés avec un Oeil sur la Resistance.

A parallel-group, cluster-randomized trial was performed in the Boboye and Loga departments of Niger from 23 November 2014 to 31 July 2017 (Fig 1). Communities were randomly selected from the same pool of communities as the main MORDOR trial in order to allow the findings of this trial to be generalizable to the parent study population (Fig 2). A set of 30 communities was randomized in a 1:1 ratio to biannual (i.e., every 6 months) mass treatment of preschool children with a single dose of either azithromycin or placebo (i.e., the same interventions offered in the main trial). Communities were followed annually with detailed morbidity assessments, including blood smears. Ethical approval was obtained from the Committee on Human Research at the University of California, San Francisco and the Institutional Review Board of the Nigerien Ministry of Health. The trial was reported according to Consolidated Standards of Reporting Trials (CONSORT) guidelines (S1 Checklist). Details of the study design were prespecified in a trial protocol (S1 Protocol).

### Participants

The unit of randomization for the trial was the grappe, a government-defined health catchment area, termed “community” in this report. Communities with a population between 200 and 2,000 on the most recent government census were eligible. No mass azithromycin distributions for trachoma had been administered in the study area for the previous 5 years, and no seasonal malaria chemoprevention programs were implemented during the study period. All children aged 1 to 59 months of age who weighed 3,800 g or more were eligible for treatment. A random selection of 40 children aged 1 to 59 months per community was invited to provide a finger-stick blood specimen for thick and thin smear. Guardians of children provided oral informed consent for both treatment and monitoring.

### Randomization and masking

Communities were randomly assigned in equal proportions to 1 of 6 letters by the trial biostatistician with the statistical package R (R Foundation for Statistical Computing, Vienna, Austria). The Nigerien study coordinator then enrolled communities and assigned the allocated intervention. Allocation was concealed at the cluster level by enrolling all communities before randomization and at the individual level by administering the treatment to all eligible children. Bottles of study drug were labelled by the manufacturer (Pfizer, New York, NY) with one of the 6 treatment letters, with 3 letters corresponding to azithromycin and 3 letters to placebo. Study bottles, packaging, and the appearance of the drug were identical. Participants, field personnel, laboratory staff, and all investigators except the trial biostatistician were masked to treatment allocation.

### Census

All households in each community were enumerated approximately every 6 months over a 2.5-year period using similar methods as the main trial [1]. All children aged ≤12 years in the household were documented.

### Monitoring

**Fig 3:**
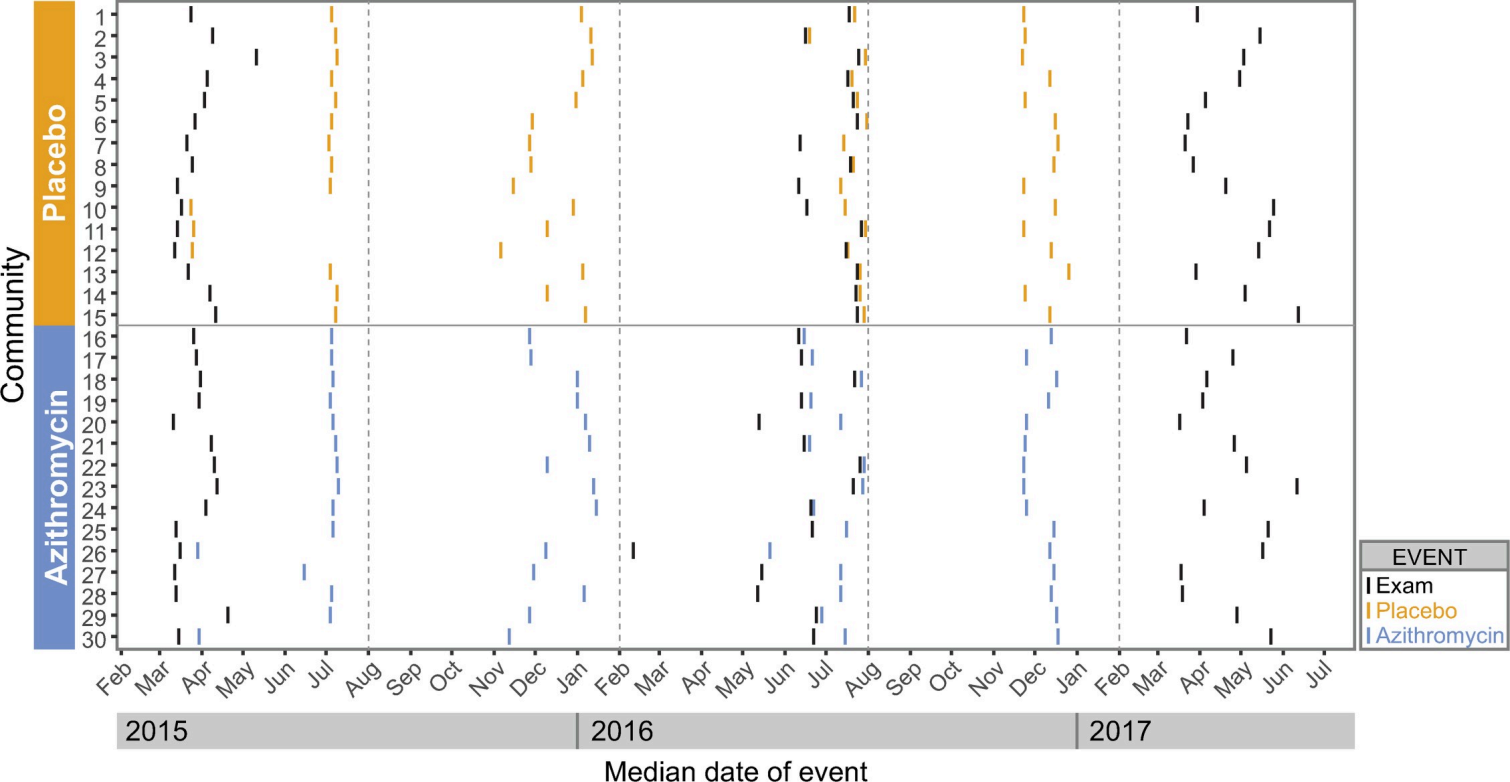
Timing of examination visits and treatment. The x-axis depicts calendar time during the trial, and the y-axis shows each community as a discrete row. Examinations and treatment visits were typically conducted over a several-day period; the vertical lines represent the median date of the examination (black) or treatment (blue for azithromycin, orange for placebo).

A monitoring visit was performed during the first census period before distribution of the study drug (median date 26 March 2015) and then approximately 12 and 24 months later (median dates 23 June 2016 and 25 April 2017, respectively). The monitoring visits at months 12 and 24 occurred before distribution of study medication for the respective study period, as well as approximately 6 months after the treatment from the previous phase. Thus, the 12-month visit was performed approximately 6 months after the second round of mass treatment, and the 24-month visit was performed approximately 6 months after the fourth round of treatment (Fig 3). As part of the assessments, each child had a finger stick performed, with hemoglobin measurements estimated with a Hemocue Hb 201^+^ device (Ängelholm, Sweden) and malaria parasitemia assessed via thick and thin smear applied to a single slide. Slides were labelled with a random number sticker to mask the laboratory personnel. After the blood had dried, the portion of the slide with the thin smear was fixed with methanol; slides were subsequently stained with 3% Giemsa. Ill-appearing children also had a drop of blood tested with a rapid diagnostic test for malaria and were referred to the local government health facility if the test was positive. The thick smear was assessed for the presence and density of parasites and gametocytes by 2 independent experienced laboratory workers at the Centre de Recherche Médicale et Sanitaire (Niamey, Niger). Parasite density, measured in parasites per microliter, was estimated as the ratio of asexual parasites to white blood cells after inspecting a minimum of 200 white blood cells, multiplied by 8,000 (i.e., an arbitrary yet accepted convention for the number of white blood cells per microliter). Thin smears were assessed for malaria species. Laboratory workers were masked to treatment allocation. Discrepancies were adjudicated by additional independent laboratory workers until a majority consensus was achieved. A consensus for density measurements required a ≤1 log_10_-unit difference between the majority of measurements; the mean density from the consensus was taken as the study measurement.

### Intervention

A single, directly observed dose of study drug was administered to all eligible children approximately every 6 months. Study treatment was administered after monitoring was complete for all children, on a different day from the monitoring visit. Field staff distributed study drug in a single community at a time. The same smartphone-based mobile application used in the census was also used for treatment. All children aged 1 to 59 months entered into the most recent census were offered treatment, and children who were absent or not eligible at the time of the previous census were entered into the mobile application and offered treatment. Azithromycin was dosed at 20 mg/kg, calculated by weight for small children and approximated by height for children who could stand [15]. The same volume of an identical-appearing placebo suspension was administered in the placebo communities. Guardians were instructed to contact a village representative if their child experienced any adverse events within 7 days of receiving study drug; the representative then informed the study coordinator. A formal adverse event survey was performed for children aged 1 to 5 months and is reported separately [16].

### Outcomes

The prespecified primary malaria outcome was the presence or absence of at least 1 parasite on thick blood smear in children 1 to 59 months of age, assessed as a community-level prevalence. Prespecified secondary outcomes included parasitemia density, gametocyte density, hemoglobin concentration at the individual level, and presence of anemia (hemoglobin < 11 g/dL) at the community level [17].

### Statistical considerations

The MORDOR study sites in Malawi, Niger, and Tanzania conducted independent trials of morbidity outcomes with slightly different methods and outcomes. In consultation with the trial’s Data Safety and Monitoring Committee, we prespecified a separate analysis for each morbidity outcome at each site (S1 Protocol). Community-level prevalence outcomes from months 12 and 24 were modeled in a mixed-effects linear regression model that included a fixed term for treatment arm, baseline prevalence, and date of sample collection and a random effect for community. Individual-level outcomes from months 12 and 24 were analyzed in mixed-effects linear or logistic regression models that included fixed effects for treatment arm and date of sample collection and nested random effects for individuals within communities. Square root transformations were used for the parasitemia prevalence, anemia prevalence, and parasite density outcomes to improve the normality of residuals. Intraclass correlation coefficients (ICCs) were derived from the regression models at the level of the randomization unit. *P* values were determined by Monte Carlo permutation (10,000 permutations). All analyses were performed in an intention-to-treat fashion with the statistical software R version 3.4.0. The trial was overseen by a Data Safety and Monitoring Committee.

### Sample size

Based on previous studies in Niger, we anticipated a prevalence of malaria parasitemia in untreated communities of 25% and an ICC of 0.056. Assuming an alpha of 0.05, enrolling 40 children in each of 15 communities per arm would provide more than 80% power to detect a 13% absolute difference between the 2 treatment arms.

## Results

Baseline characteristics of the 15 azithromycin-treated communities and 15 placebo-treated communities are shown in Table 1. Communities in the placebo group were on average larger than those in the azithromycin group, with a total of 1,695 children aged 1 to 59 months enumerated in the azithromycin arm and a total of 3,029 children enumerated in the placebo arm at baseline. The age and sex distribution within communities was similar between the 2 groups. All communities received their allocated study medication, and none were lost to follow-up. Across all 4 study visits, study drug was distributed to 78.7% (95% CI 74.9%–82.6%) of eligible children in the azithromycin group and 81.7% (95% CI 78.9%–84.5%) of eligible children in the placebo group (Fig 1).

**Table 1:** Baseline characteristics of the study communities, as assessed from a population census.

Characteristic	Mean (95% CI)	
	Placebo *N* = 15	Azithromycin *N* = 15
**Census**		
**No. of children 1–59 mo**	202 (136–268)	113 (63–163)
**Age, %**		
0 y	14.6% (11.9%–17.2%)	13.8% (11.3%–16.4%)
1 y	14.4% (12.3%–16.6%)	15.2% (12.4%–18.0%)
2 y	19.3% (17.5%–21.1%)	18.8% (16.0%–21.6%)
3 y	23.8% (21.4%–26.3%)	24.6% (21.7%–27.5%)
4 y	27.9% (24.1%–31.7%)	27.5% (24.3%–30.7%)
**Female, %**	48.0% (45.7%–50.3%)	48.2% (45.5%–50.8%)

No hospitalizations or life-threatening illnesses were reported in either group over the duration of the study. Guardians of 1- to 5-month-old children were surveyed about adverse events approximately 1 month after treatment given the paucity of azithromycin safety data in this population. Detailed results have been published elsewhere [16]. In summary, the most common guardian-reported adverse events were as follows: diarrhea, which was reported in 110 out of 571 (19.3%) children in the azithromycin group and 321 out of 1,141 (28.1%) children in the placebo group (*P* = 0.03); vomiting, reported in 91 out of 571 (15.9%) from the azithromycin group and 240 out of 1,141 (21.0%) from the placebo group (*P* = 0.07); and rash, reported in 70 out of 571 (12.3%) from the azithromycin group and 155 out of 1,141 (13.6%) from the placebo group (*P* = 0.07).

**Fig 4:**
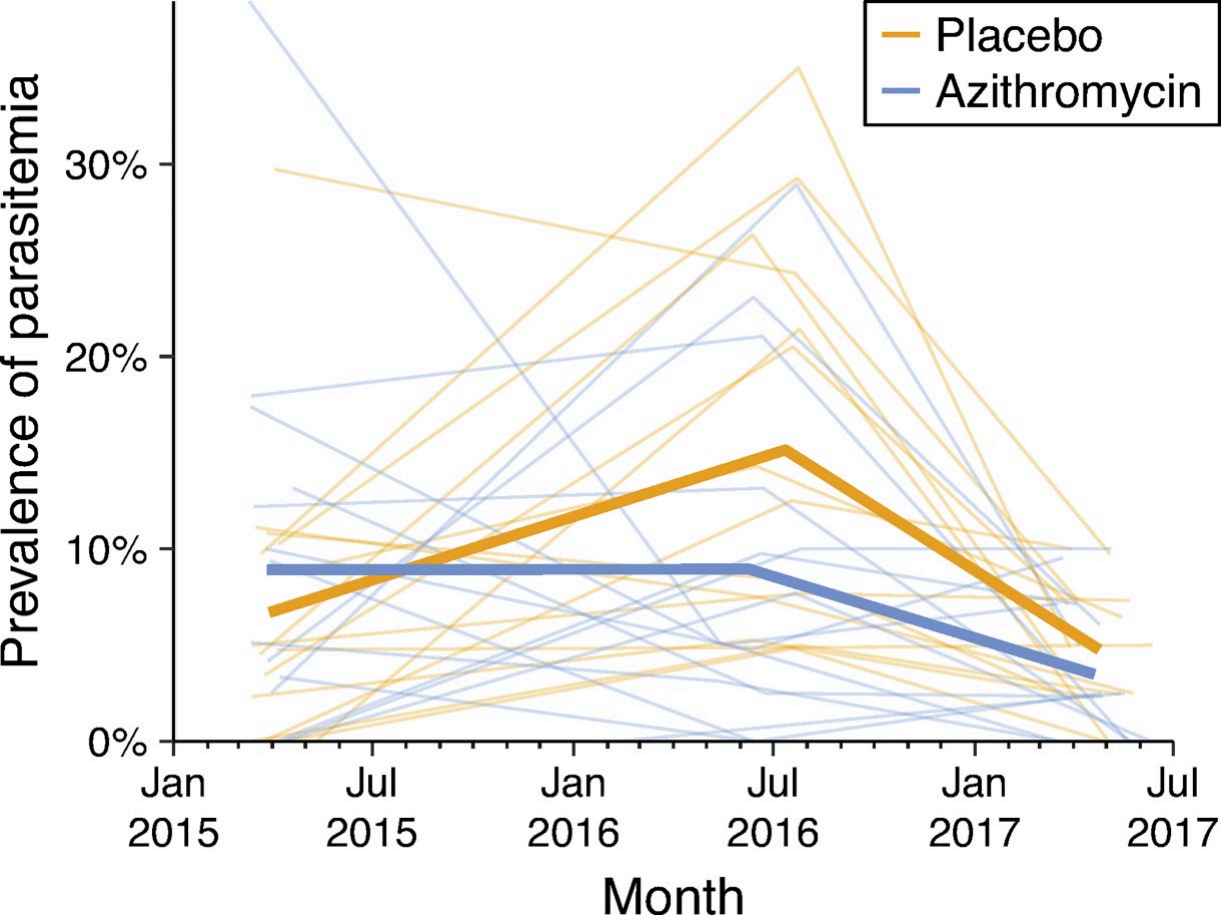
Community-specific malaria parasitemia prevalence among 1- to 59-month-old children at the 3 annual monitoring visits. Each thin line represents a community, and the thick lines represent the mean prevalence of parasitemia in each trial arm.

The prevalence of parasitemia among 1- to 59-month-old children for each community over time is shown in Table 2 and Fig 4. At baseline, the mean prevalence of malaria parasitemia was 6.7% (95% CI 4.0%–12.6%) in the placebo group and 8.9% (95% CI 5.1%–15.7%) in the azithromycin group. At month 12, the mean prevalence was 15.3% (95% CI 10.8%–20.6%) and 8.8% (95% CI 5.1%–14.3%) in the placebo and azithromycin groups, respectively, and at month 24, these estimates were 4.8% (3.3%–6.4%) and 3.5% (1.9%–5.5%), respectively. Parasitemia prevalence was significantly lower in the azithromycin group at months 12 and 24 after adjusting for baseline parasitemia and the date of sample collection (*P* = 0.02; ICC = 0.26; square-root–transformed outcome; prespecified primary outcome). If the mean malaria prevalence was 10% in the placebo-treated communities (i.e., the post-treatment average observed in the present study), then azithromycin would be predicted to reduce the prevalence of malaria parasitemia by an absolute difference of 5.3% (95% CI −1.4 to −8.0%). The conclusions did not change in a sensitivity analysis of individual-level data (odds ratio [OR] 0.54 relative to the placebo group, 95% CI 0.30–0.97, adjusted for date of sample collection).

**Table 2:** Community-specific prevalence of malaria parasitemia.

	No. of positive out of total tested, per community (%)					
Community	Month 0		Month 12		Month 24	
**Placebo**						
**1**	0/40	(0%)	5/40	(12.5%)	4/40	(10.0%)
**2**	2/23	(8.7%)	4/28	(14.3%)	2/31	(6.5%)
**3**	0/38	(0%)	6/28	(21.4%)	0/42	(0%)
**4**	2/42	(4.8%)	2/41	(4.9%)	0/41	(0%)
**5**	11/37	(29.7%)	9/37	(24.3%)	3/41	(7.3%)
**6**	4/37	(10.8%)	3/36	(8.3%)	1/42	(2.4%)
**7**	1/22	(4.5%)	5/19	(26.3%)	1/25	(4.0%)
**8**	1/29	(3.4%)	8/39	(20.5%)	3/42	(7.1%)
**9**	1/43	(2.3%)	2/38	(5.3%)	1/44	(2.3%)
**10**	4/36	(11.1%)	3/40	(7.5%)	1/40	(2.5%)
**11**	2/40	(5.0%)	4/40	(10.0%)	3/41	(7.3%)
**12**	0/37	(0%)	2/40	(5.0%)	1/41	(2.4%)
**13**	4/41	(9.8%)	14/40	(35.0%)	2/41	(4.9%)
**14**	4/39	(10.3%)	12/41	(29.3%)	4/41	(9.8%)
**15**	0/38	(0%)	2/41	(4.9%)	2/40	(5.0%)
***Mean (95% CI)***	*6*.*7% (4*.*0%–12*.*6%)*		*15*.*3% (10*.*8%–20*.*6%)*		*4*.*8% (3*.*3%–6*.*4%)*	
**Azithromycin**						
**16**	4/40	(10.0%)	2/42	(4.8%)	4/42	(9.5%)
**17**	1/24	(4.2%)	9/39	(23.1%)	2/33	(6.1%)
**18**	1/40	(2.5%)	11/38	(28.9%)	2/40	(5.0%)
**19**	3/32	(9.4%)	0/28	(0%)	0/21	(0%)
**20**	15/39	(38.5%)	2/39	(5.1%)	0/41	(0%)
**21**	1/30	(3.3%)	0/42	(0%)	1/40	(2.5%)
**22**	0/40	(0%)	4/40	(10.0%)	4/40	(10.0%)
**23**	0/39	(0%)	3/39	(7.7%)	0/40	(0%)
**24**	0/41	(0%)	4/41	(9.8%)	3/42	(7.1%)
**25**	7/39	(17.9%)	8/38	(21.1%)	0/40	(0%)
**26**	0/26	(0%)	0/16	(0%)	1/40	(2.5%)
**27**	8/46	(17.4%)	1/39	(5.0%)	3/42	(7.1%)
**28**	2/37	(5.4%)	1/32	(3.1%)	0/23	(0%)
**29**	5/38	(13.2%)	1/40	(2.5%)	1/43	(2.3%)
**30**	5/41	(12.2%)	5/38	(13.2%)	0/40	(0%)
***Mean (95% CI)***	*8*.*9% (5*.*1%–15*.*7%)*		*8*.*8% (5*.*1%–14*.*3%)*		*3*.*5% (1*.*9%–5*.*5%)*	

The average parasite density among parasitemic children is shown for each treatment arm in Table 3. Parasite density was lower in the azithromycin group in a mixed-effects linear regression of the 12- and 24-month values adjusted for the date of sample collection, with density estimates 7,540 parasites/μl lower (95% CI −350 to −12,550 parasites/μl) than the placebo arm assuming a mean parasite density in the placebo arm of 17,000 as observed in this study (ICC = 0.02; *P* = 0.02; square-root–transformed outcome). No significant differences in gametocyte prevalence or density were observed between the 2 groups (S1 Table, S2 Table).

**Table 3:** Individual-level parasite density and hemoglobin.

	Parasite density, parasites/μl*				Hemoglobin, g/dL			
	Placebo		Azithromycin		Placebo		Azithromycin	
Month	*N*	Mean (95% CI)	*N*	Mean (95% CI)	*N*	Mean (95% CI)	*N*	Mean (95% CI)
**0**	36	550 (250–1,200)	52	70,020 (600–225,890)	542	9.7 (9.5–10.0)	552	9.8 (9.5–10.0)
**12**	81	22,470 (4,390–56,360)	51	400 (250–760)	548	9.1 (8.7–9.5)	551	9.7 (9.2–10.2)
**24**	28	740 (150–2,370)	21	640 (130–1,535)	592	10.1 (9.9–10.3)	567	10.2 (9.9–10.4)

*Among parasitemic children; rounded to nearest 10.

Values represent individual-level means with bootstrapped 95% CIs resampled at the community level.

Mean hemoglobin measurements are summarized for each study visit and treatment arm in Table 3. No significant difference was observed between the 2 groups after adjusting for date of sample collection, although the azithromycin group had slightly higher hemoglobin measurements (mean 0.34 g/dL higher than the placebo group, 95% CI −0.06 to 0.75 g/dL; ICC = 0.09; *P* = 0.10). Similarly, the prevalence of anemia was not significantly different between the 2 groups in the permutation test (*P* = 0.06; ICC = 0.08; square-root–transformed outcome; S3 Table), although the regression model predicted less anemia in the azithromycin-treated communities (absolute difference 6.7%, 95% CI −0.2 to −12.8%, assuming a 75% prevalence of anemia as approximately observed in the placebo-treated communities).

## Discussion

In this placebo-controlled study, mass azithromycin distributions resulted in a significantly lower prevalence of malaria parasitemia and lower parasite density levels when assessed at 2 annual post-treatment study visits. Hemoglobin levels were slightly greater and the prevalence of anemia slightly lower in azithromycin-treated communities, although neither of these were statistically significant. These results raise the possibility that reductions in malaria may in part explain the childhood mortality benefit of azithromycin.

The prevalence of malaria parasitemia was lower in this study than in several other population-based studies done in other parts of Niger [9, 13]. This may be explained by geographic and year-to-year variations in malaria prevalence [18–20]. The relatively low malaria prevalence could also be due to the timing of the annual monitoring visits, which were done in the spring, before the major seasonal peak in the fall. The prevalence of malaria would be expected to increase later in the summer. Indeed, the malaria estimates were higher in each group at the month 12 assessments, which on average occurred several months later than at the other 2 time points. The preponderance of malaria during the autumn months in Niger was suggested by the parent MORDOR trial since the largest number of childhood deaths occurred during this time and malaria was the most common attributed cause of death from verbal autopsies, although it is important to note that verbal autopsy has poor diagnostic accuracy for malarial deaths and can lead to overestimates in areas with endemic malaria like Niger [1, 21, 22]. The strength of the statistical association between mass azithromycin treatments and malaria could depend on the timing of treatments as well as the timing of assessments, so it is possible that the present trial did not maximize the chance of finding an antimalarial effect of azithromycin. Nonetheless, our finding of reduced parasitemia in the azithromycin group even in the presumably low-prevalence spring months is consistent with azithromycin exerting a sustained reduction in malarial burden throughout the year.

The present study is consistent with several previous studies that have assessed the antimalarial activity of azithromycin. As is the case with some other antibiotics, azithromycin acts against the parasite apicoplast and exerts slow antimalarial activity [6]. In clinical trials, azithromycin has been found to improve the treatment efficacy of both artesunate and chloroquine relative to each agent alone [4, 5, 11]. Azithromycin has also been shown to prevent malaria infections, although its efficacy appears to be inferior to that of doxycycline [2, 3]. Malaria outcomes have been measured during studies of mass azithromycin distributions for trachoma, with reductions in malaria parasitemia observed in some studies but not in others [9, 13, 23, 24]. The present study improved upon previous studies of mass azithromycin distribution in that it was placebo controlled and enforced strict masking procedures both in the field and by laboratory staff.

The public health impact of MORDOR remains to be seen. The mortality benefit of mass azithromycin distributions was especially strong for the youngest children and for the Niger site, so it may ultimately make sense to target antibiotic distributions to those most likely to benefit. Doing so would have the added benefit of reducing the total volume of antibiotics distributed, which may limit antimicrobial resistance. Studies like this one may help elucidate where to target antibiotics. Namely, our results suggest that mass azithromycin distributions might be more effective in reducing mortality in places with prevalent malaria. Subsequent studies assessing the impact of mass azithromycin on other common causes of childhood mortality will be important, as will studies in areas without prevalent malaria. For example, if the mortality benefit of mass azithromycin is mediated only through malaria reduction, then seasonal malaria chemoprevention might be a more efficient way to prevent childhood mortality. In fact, a recent household-randomized trial found that adding azithromycin to seasonal malaria chemoprevention did not provide a benefit for malaria or mortality, perhaps because any antimalarial effect of azithromycin was reduced when given along with the more effective antimalarial drugs [25]. However, that same study also found that azithromycin treatment was associated with reductions in diarrheal and respiratory infections. If other studies confirm these associations, wider implementation of mass azithromycin distributions may be warranted.

Although the most likely explanation for the observed result is a direct effect of azithromycin on those children who took the drug, it is important to note that mass antibiotics may also have indirect spillover effects [14]. By reducing the overall burden of parasitemia, mass azithromycin distributions reduced the reservoir of parasites in the community, which may have supplemented any direct effect of the antibiotic.

The present trial has several limitations. Study drug was generally administered in the dry seasons. Although mathematical models have suggested that this may in fact be the optimal time to reduce the community burden of malaria, the ideal time to administer antibiotics remains unclear [26]. Likewise, due to logistical reasons, monitoring visits were conducted in the dry season, which may not provide a complete picture of the impact of the intervention during the peak malaria transmission season. Grading of thick smears is inherently subjective, although the possibility of misclassification error was mitigated by having at least 2 independent laboratory staff assess each slide. Finally, the generalizability of the results outside Niger remains unclear, and even the generalizability within Niger is not assured given considerable variation in malaria transmission within the country [18–20]. The other 2 MORDOR sites in Malawi and Tanzania had a lower reduction of mortality in subgroup analyses, and thus the malaria results for these sites will provide important context for the interpretation of the present report and may ultimately alter our understanding of the causal relationship between mass azithromycin, malaria, and mortality.

In conclusion, a placebo-controlled, cluster-randomized trial found that biannual mass azithromycin distributions targeted to preschool children in Niger resulted in a reduction in malaria parasitemia in 1- to 59-month-old children. Study communities were selected from the same set of communities and treated with the same intervention as the parent MORDOR trial, so results of this study can be extrapolated to the larger trial. As such, this study suggests that the mortality benefits conveyed by mass azithromycin distributions may have been in part due to the antimalarial activity of azithromycin. Similar trials performed in other settings will be important to confirm the association found in this trial.

## Supporting information

S1 Checklist Consolidated standards of reporting trials (CONSORT) checklist. (DOCX)

S1 Table Community-level gametocyte prevalence. (DOCX)

S2 Table Individual-level gametocyte density. (DOCX)

S3 Table Community-specific prevalence of anemia. (DOCX)

S1 Protocol Manual of procedures and statistical analysis plan for MORDOR. MORDOR, Macrolides Oraux pour Réduire les Décés avec un Oeil sur la Resistance.
(PDF)

S1 Data Malaria and anemia outcomes. (CSV)

## Abbreviations

CONSORT: Consolidated Standards of Reporting Trials
ICC: intraclass correlation coefficient
MORDOR: Macrolides Oraux pour Réduire les Décés avec un Oeil sur la Resistance
OR: odds ratio
