# Supplementary material for: Biannual mass azithromycin distributions and malaria parasitemia in pre-school children in Niger: A cluster-randomized, placebo-controlled trial
Source: PLoS Med. 2019 Jun 25;16(6):e1002835. doi: 10.1371/journal.pmed.1002835 (PMC6592520; doi:10.1371/journal.pmed.1002835)
Supplement: S1 Table — (DOCX) [file pmed.1002835.s002.docx]

**S1 Table. Community-level gametocyte prevalence**

|  | Placebo | |  | Azithromycin | |
| --- | --- | --- | --- | --- | --- |
| Study visit | N | Mean (95%CI) |  | N | Mean (95%CI) |
| Month 0 | 15 | 0.4% (0 to 1.1%) |  | 15 | 0.2% (0 to 0.5%) |
| Month 12 | 15 | 0.3% (0 to 0.8% |  | 15 | 0% (0 to 0.4%) |
| Month 24 | 15 | 0.3% (0 to 0.8%) |  | 15 | 0.2% (0 to 0.5%) |
| * P=0.27 comparing treatment arms at months 12 and 24 in linear mixed effects regression adjusted for baseline gametocyte prevalence and including community as a random effect; 0.2% lower in azithromycin arm (95%CI -0.6 to 0.2%; intraclass correlation coefficient=0.27. | | | | | |
