# Supplementary material for: Biannual mass azithromycin distributions and malaria parasitemia in pre-school children in Niger: A cluster-randomized, placebo-controlled trial
Source: PLoS Med. 2019 Jun 25;16(6):e1002835. doi: 10.1371/journal.pmed.1002835 (PMC6592520; doi:10.1371/journal.pmed.1002835)
Supplement: S2 Table — (DOCX) [file pmed.1002835.s003.docx]

**S2 Table. Individual-level gametocyte density (parasites per** $\boldsymbol{\mu}\boldsymbol{L}^{\boldsymbol{2}}$**)^†^**

|  | Placebo | |  | Azithromycin | |
| --- | --- | --- | --- | --- | --- |
| Study visit | N | Mean (Range) |  | N | Mean(Range) |
| Month 0 | 2 | 95 (60 to 130) |  | 1 | 400 |
| Month 12 | 2 | 51 (1 to 100) |  | 0 | --- |
| Month 24 | 2 | 1 (1 to 1) |  | 1 | 1 |
| ^†^ Among children with parasitemia; P=0.97 comparing treatment arms at months 12 and 24 in mixed effects linear regression with community as a random effect; 1.5 lower in azithromycin arm (95%CI -93 to 90; intraclass correlation coefficient<0.001. | | | | | |
