# Supplementary material for: Biannual mass azithromycin distributions and malaria parasitemia in pre-school children in Niger: A cluster-randomized, placebo-controlled trial
Source: PLoS Med. 2019 Jun 25;16(6):e1002835. doi: 10.1371/journal.pmed.1002835 (PMC6592520; doi:10.1371/journal.pmed.1002835)
Supplement: S3 Table — (DOCX) [file pmed.1002835.s004.docx]

**S3 Table. Community-specific prevalence of anemia**

|  | No. with hemoglobin <11 g/dL / total tested, per community (%) | | | | | |
| --- | --- | --- | --- | --- | --- | --- |
| Community | Month 0 | | Month 12 | | Month 24 | |
| Placebo |  |  |  |  |  |  |
| 1 | 33/40 | (82.5%) | 36/40 | (90.0%) | 27/40 | (67.5%) |
| 2 | 16/23 | (69.6%) | 23/28 | (82.1%) | 19/31 | (61.3%) |
| 3 | 27/38 | (71.1%) | 26/28 | (92.9%) | 29/42 | (69.0%) |
| 4 | 28/42 | (66.7%) | 37/41 | (90.2%) | 22/41 | (53.7%) |
| 5 | 26/37 | (70.3%) | 37/37 | (100%) | 29/41 | (70.7%) |
| 6 | 32/37 | (86.5%) | 31/36 | (86.1%) | 24/42 | (57.1%) |
| 7 | 20/22 | (90.9%) | 16/19 | (84.2%) | 17/25 | (68.0%) |
| 8 | 27/29 | (93.1%) | 31/39 | (79.5%) | 28/42 | (66.7%) |
| 9 | 37/43 | (86.0%) | 21/38 | (55.3%) | 28/44 | (63.6%) |
| 10 | 26/36 | (72.2%) | 37/40 | (92.5%) | 36/40 | (90.0%) |
| 11 | 26/40 | (65.0%) | 31/40 | (76.9%) | 31/41 | (75.6%) |
| 12 | 25/37 | (67.6%) | 35/40 | (87.5%) | 28/41 | (68.3%) |
| 13 | 36/41 | (87.8%) | 36/40 | (90.0%) | 31/41 | (75.6%) |
| 14 | 33/39 | (84.6%) | 34/41 | (82.9%) | 29/41 | (70.7%) |
| 15 | 28/38 | (73.7%) | 34/41 | (82.9%) | 32/40 | (80.0%) |
| *Mean (95%CI)* | *77.8% (73.3-8289%)* | | *84.9% (77.8-88.6%)* | | *69.2% (65.2-74.0%)* | |
| Azithromycin |  |  |  |  |  |  |
| 16 | 35/40 | (87.5%) | 26/42 | (61.9%) | 29/42 | (69.0%) |
| 17 | 18/24 | (75.0%) | 25/39 | (64.1%) | 21/33 | (63.6%) |
| 18 | 28/40 | (70.0%) | 33/38 | (86.8%) | 28/40 | (70.0%) |
| 19 | 28/32 | (87.5%) | 16/28 | (57.1%) | 19/21 | (90.5%) |
| 20 | 33/39 | (84.6%) | 33/39 | (84.6%) | 30/41 | (73.2%) |
| 21 | 21/30 | (70.0%) | 28/42 | (66.7%) | 20/40 | (50.0%) |
| 22 | 30/40 | (75.0%) | 28/40 | (70.0%) | 28/40 | (70.0%) |
| 23 | 30/39 | (76.9%) | 39/39 | (100%) | 31/40 | (77.5%) |
| 24 | 27/41 | (65.9%) | 21/41 | (51.2%) | 29/42 | (69.0%) |
| 25 | 31/39 | (79.5%) | 33/38 | (86.8%) | 30/40 | (75.0%) |
| 26 | 21/26 | (80.8%) | 10/16 | (62.5%) | 32/40 | (80.0%) |
| 27 | 35/46 | (76.1%) | 26/39 | (66.7%) | 28/42 | (66.7%) |
| 28 | 17/37 | (45.9%) | 16/32 | (50.0%) | 11/23 | (47.8%) |
| 29 | 28/38 | (73.7%) | 31/40 | (77.5%) | 33/43 | (76.7%) |
| 30 | 29/41 | (70.7%) | 29/38 | (76.3%) | 24/40 | (60.0%) |
| *Mean (95%CI)* | *74.6% (68.0-78.4%)* | | *70.8% (64.3-78.5%)* | | *69.3% (63.4-74.4%)* | |
